# Improving an Intelligent Detection System for Coronary Heart Disease Using a Two-Tier Classifier Ensemble

**DOI:** 10.1155/2020/9816142

**Published:** 2020-04-27

**Authors:** Bayu Adhi Tama, Sun Im, Seungchul Lee

**Affiliations:** ^1^Department of Mechanical Engineering, Pohang University of Science and Technology, Republic of Korea; ^2^Department of Rehabilitation Medicine, Bucheon St. Mary's Hospital, College of Medicine, The Catholic University of Korea, Republic of Korea

## Abstract

Coronary heart disease (CHD) is one of the severe health issues and is one of the most common types of heart diseases. It is the most frequent cause of mortality across the globe due to the lack of a healthy lifestyle. Owing to the fact that a heart attack occurs without any apparent symptoms, an intelligent detection method is inescapable. In this article, a new CHD detection method based on a machine learning technique, e.g., classifier ensembles, is dealt with. A two-tier ensemble is built, where some ensemble classifiers are exploited as base classifiers of another ensemble. A stacked architecture is designed to blend the class label prediction of three ensemble learners, i.e., random forest, gradient boosting machine, and extreme gradient boosting. The detection model is evaluated on multiple heart disease datasets, i.e., Z-Alizadeh Sani, Statlog, Cleveland, and Hungarian, corroborating the generalisability of the proposed model. A particle swarm optimization-based feature selection is carried out to choose the most significant feature set for each dataset. Finally, a two-fold statistical test is adopted to justify the hypothesis, demonstrating that the performance differences of classifiers do not rely upon an assumption. Our proposed method outperforms any base classifiers in the ensemble with respect to 10-fold cross validation. Our detection model has performed better than current existing models based on traditional classifier ensembles and individual classifiers in terms of accuracy, *F*_1_, and AUC. This study demonstrates that our proposed model adds a considerable contribution compared to the prior published studies in the current literature.

## 1. Introduction

Effective detection and diagnosis of coronary heart disease (CHD) are compulsory to prevent human casualties. CHD is the most common type of heart disease, and it accounts for 37,000 deaths annually in the United States in 2015 [1]. The factors that increase a person's risk can be prevalently lifestyle-related elements, i.e., hypertension, cholesterol, obesity, and smoking. However, some of the nonlifestyle risk factors, i.e., family history, age, and having high levels of *fibrinogen* must also be taken into consideration. Moreover, it develops without any risk factors as mentioned above, which may lead to a heart attack without causing any prior apparent symptoms. Hence, CHD is one of the leading cardiovascular diseases with a high mortality rate, making it one of the complicated diseases to treat.

In order to examine the suspicious sign of CHD, particular tests might be required by a physician such as *angiogram*, blood test, blood pressure monitoring, chest X-ray, electrocardiogram, echocardiogram (heart ultrasound), and stress tests [2, 3]. The electrocardiogram, which represents the electrical activity of the heart, is one of the standards and noninvasive diagnostic tests in CHD. Though it can be performed rapidly and is easy to perform, it may miss out the asymptomatic patients and diagnose them with normal electrocardiogram rhythm. Also, the electrocardiogram has some limitations as a prognostic tool to predict future CHD [4, 5]. This disadvantage gives rise to *angiogram*, which is a rule of thumb for disease detection and diagnosis. Nevertheless, it is uneconomical and calls for specialized technical competence. As a result, many practitioners/researchers nowadays are soliciting more economical and efficient approaches using machine learning for diagnosing CHD.

Intelligent systems have been deployed in clinical-based decision support systems, assisting physicians in providing a second opinion about the detection and diagnosis of particular diseases [6]. Due to the fact that CHD might be challenging to address, inaccurate detection or delays in clinical treatment might lead to a poor outcome or increased mortality. CHD detection is dependent on lots of variables such as family history, age, and gender, to name a few [7]. Furthermore, it varies on the detection methods used and the variables chosen. Artificial intelligence (AI) and machine learning techniques have brought a new magnitude to CHD detection and diagnosis. They have been employed for discovering and uncovering valuable pattern/information from the clinical datasets with a few user inputs and attempts [8, 9].

By its nature, clinical datasets are uncertain and irregular; thus, it is not straightforward to apply machine learning techniques without an adequate preprocessing task, e.g., feature selection. Moreover, data irregularities in a medical dataset are deemed to have an effect on the final performance of the classification model [10]. Therefore, in order to achieve the maximum capability of machine learning algorithms, it is essential to take into consideration a proper data preparation technique. Moreover, some unnecessary features might degrade the performance of algorithms; thus, having a data preparation and feature selection are compulsory to gain the best possible accuracy in predicting CHD. Not-withstanding the fact that a feature selection technique is equally crucial with the choice of a proper technique, it is still not apparent on how to combine machine learning techniques with a suitable feature set. The issue depicts us that there exists an open research problem in identifying the merit of the feature set and in picking an appropriate classification algorithm.

Many researchers have considered different kinds of classifiers for predicting CHD, either as individual classifiers or meta classifiers. In the case of an individual classifier that cannot give a desirable performance, a meta (e.g., ensemble) classifier should be accommodated to provide a significant improvement over individual classifiers. Unlike single classifiers, meta classifiers train multiple classifiers to predict the final prediction outcome, making them robust and sufficient for disease prediction. The choice of combining multiple classifiers can be either homogeneous (e.g., using the same type of classifiers) or heterogeneous (e.g., using different types of classifiers). Although in many other application domains, meta classifiers have shown remarkable performance over individual classifiers; choosing a variety of combination techniques and base classifiers remains unexplored [11].

## 2. Related Work

Most existing CHD prediction techniques have been built and validated on UCI Machine Learning Repository datasets, which are composed of risk factors (e.g., variables) excluding *angiography*. These techniques are simpler, less expensive, replicable, and unbiased diagnoses and can detect automatically and can perform a preliminary examination of patients based on clinical data in hospitals. In this section, we summarize machine learning that uses risk factors for training and testing the classification models, particularly on the datasets available on the UCI website. The two-tier ensemble presented in this paper is also validated on those datasets. However, various types of methods, risk factors, and datasets have been proposed for CHD diagnosis [12]. A well-known ensemble learning, namely, rotation forest with different base classifiers was assessed [13]. Based on the performance validation on the Cleveland dataset, rotation forest with RBF network as base classifier was the top-performing classifier. The work of Muthukaruppan and Er [14] presented a PSO-based fuzzy expert system for the diagnosis of CHD. Rules were extracted from decision tree, and they were converted into fuzzy rules. Having PSO to tune the fuzzy membership function, the fuzzy expert system yielded 93.27% accuracy on the Cleveland dataset. The potential of an expert judgment-based feature selection was explored in [15]. Using 10-fold cross validation (10CV) for evaluation, sequential minimal optimization (SMO) was the best performer on the Cleveland dataset.

A work of Alizadehsani et al. [16] took into account an ensemble approach, namely, Bagging-C4.5, for CHD prediction. The proposed classifier reached accuracy rates of 79.54%, 61.46%, and 68.96% for the diagnosis of the stenoses of the left anterior descending (LAD), left circumflex (LCX), and right coronary artery (RCA), respectively. A dataset collected from Rajaie Cardiovascular Medical and Research Center, having 54 input features and 303 instances, was used in the experiment. Similar authors in [17] used several machine learning algorithms such as Bagging, SMO, neural network (NN), and naive Bayes. The best accuracy was achieved by SMO at 94.08%. An information gain-based feature selection was also involved in choosing a suitable feature set. Moreover, Alizadehsani et al. [18] aimed at improving the accuracy in the diagnosis of the stenosis of each major coronary artery. To achieve this, the authors proposed a feature selection to choose more discriminative feature subsets for each artery. Based on their experiment, the proposed classifier, e.g., support vector machine (SVM) gained accuracy rates at 86.14%, 83.17%, and 83.50% for LAD, LCX, and RCA, respectively. A novel hybrid approach for CHD diagnosis based on the combination of CFS, PSO, and *K*-means clustering was initiated in [19]. The proposed model is tested on Cleveland and IGMC datasets, having 83.5% and 90.28% accuracy, respectively. A study presented by Qin et al. [20] incorporated multiple feature selection methods into the ensemble algorithm to verify the importance of feature selection in the Z-Alizadeh Sani CHD dataset. Weight optimization of NN via the genetic algorithm used for heart disease detection was introduced in Arabasadi et al. [21]. The proposed classifier was tested on the Z-Alizadeh Sani dataset, obtaining 93.85%, 97%, and 92% in terms of accuracy, sensitivity, and specificity, respectively. A research of Haq et al. [22] proposed a hybrid feature selection and logistic regression to classify heart disease, while Dwivedi [23] evaluated the performance of several machine learning algorithms for heart disease prediction. Logistic regression was reported as the best classifier, providing 85% accuracy on the Statlog dataset. Furthermore, the performance of boosted C5.0 and NN were compared to predict CHD for the Cleveland dataset [24]. Based on the experiment, the authors concluded that there was no significant difference between C5.0 and NN.

More recently, Abdar et al. [25] established a new optimization technique called N2Genetic optimizer. The nuSVM was then used to classify the patients having CHD or not. The proposed detection method was compared against existing works, yielding accuracy at 93.08% on the Z-Alizadeh Sani dataset. An ensemble architecture using majority voting was suggested by Raza [26]. It combined logistic regression, multilayer perceptron, and naive Bayes to predict heart disease in a patient. Classification accuracy of 88.88% was achieved, where it was better than any individual base classifiers. Similarly, Amin et al. [9] attempted to seek the best appropriate features for CHD diagnosis. A voting-based ensemble of naive Bayes and logistic regression was utilized for training the selected feature subset of 9 features of the Cleveland dataset. The final predictive performance was achieved by 87.41% with respect to 10CV approach. Most recently, Mohan et al. [27] proposed a hybrid method for heart disease prediction based on the combination of random forest with a linear model (HRFLM). The proposed method enhanced the performance level with an accuracy of 88.7% on the Cleveland dataset. Based on our discussion as mentioned earlier, we chronologically summarize existing works, as shown in Table 1. From the abovementioned evidences, existing CHD detection methods suffer from some of the following shortcomings: firstly, most researchers have validated their proposed method on a particular dataset and only a few works have used at least two CHD datasets in their experiments, i.e., [9, 19, 21, 27]. This makes the prediction results not reliable enough. It is highly desirable to use multiple CHD datasets in order to prove the generalizability of the proposed method.

Secondly, the absence of a statistical significance test is the key drawback of prior works. According to Demˇsar [28], a significance test is a plausible approach to compare multiple classification algorithms and multiple datasets. Since there exists no such test, any significant differences among classification algorithms are still questionable and inestimable. Lastly, some existing works have taken into account classifier ensembles, i.e., [9, 26]; however, the classifier ensembles are constructed based on several weak individual classifiers. i.e., decision tree, neural network, and logistic regression, to name a few; thus, the final prediction outcome could not be gained.

To cope with those limitations, the objective of this study is to design a two-tier classifier ensemble to predict heart disease. The proposed ensemble learner is built based on a stacked architecture, in which its base classifiers are taken from other types of classifier ensembles, i.e., random forest [29], gradient boosting machine [30], and extreme gradient boosting (XGBoost) [31]. Also, an experiment is carried out to identify the most significant features using a combination of feature subset selection, e.g., correlation feature selection (CFS) [32] and optimization technique, e.g., particle swarm optimization (PSO). Heart disease datasets are obtained from a public resource, namely, the UCI Machine Learning Repository. These include Z-Alizadeh Sani [21], Statlog [33], Cleveland [34], and Hungarian dataset [34]. In addition, we conduct a two-step statistical significance test in order to assess how significant the performance differences among classifiers are. Finally, this study benchmarks the performance accuracy achieved by the proposed classifier against the best accuracy obtained in the existing literature.

## 3. Materials and Methods

This section provides the materials (e.g., datasets) and methods used in our experiment. It consists of details about datasets, a conceptual workflow of heart disease detection, feature selection, and the classification techniques, i.e., random forest, gradient boosting machine, extreme gradient boosting machine, and the proposed two-tier ensemble.

### 3.1. Heart Disease Datasets

Datasets considered for heart disease prediction are obtained from generally accessible repository [35]. These datasets are chosen because other researchers in this field frequently utilize them. The following are the outline of the datasets used in the experiment, while Table 2 summarizes each dataset's characteristics and properties.

#### 3.1.1. Z-Alizadeh Sani

The dataset includes 303 patients, where 216 of whom have CHD. Fifty-five input variables and a class label variable are collected from each patient. The variables incorporate some of the patient's characteristics, such as demographic, symptom and examination, electrocardiography, and laboratory examinations [21]. In the original dataset, the class label variable is comprised of four categories, i.e., normal, LAD, LCX, and RCA. Since our aim is to solve a binary classification problem, we group LAD, LCX, RCA into CHD category, supposing that there are two classes in the class label attribute.

#### 3.1.2. Statlog

In its original version, the dataset is made up of 75 attributes. However, many researchers have used 13 attributes for CHD detection. No missing values exist, and 261 instances were successfully collected, in which 114 patients have suffered from CHD [36].

#### 3.1.3. Cleveland

The dataset is collected by Detrano et al. [34] from 303 samples of normal and CHD patients. The original dataset consists of 76 variables; however, we consider 13 variables as other prior works did. The class label attribute is normalized into two distinct classes, i.e., yes (the presence of CHD) and no (the absence of CHD) because in the original dataset, five integer values ranging from 0 (no CHD) to 4 (severe CHD) exist.

#### 3.1.4. Hungarian

We consider a processed dataset available in the UCI repository. The 210 dataset includes 13 input features and a total of 294 observations. Moreover, 106 patients are identified as CHD sufferers, while the rest are in normal condition (CHD is not found).

### 3.2. Framework of Heart Disease Detection

**Figure 1:**
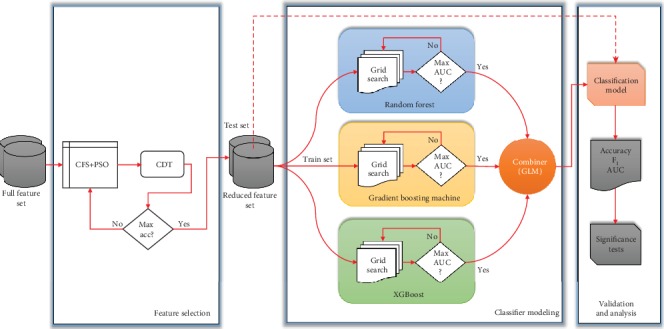
Theoretical framework of heart disease detection.

A conceptual framework of CHD detection is visualized in Figure 1. The workflow is made up of three phases, i.e., feature selection, classifier modeling, and validation analysis. The first phase deals with the procedure of precisely determining a set of features as the most relevant for CHD detection at hand. It is carried out by employing a correlation-based feature selection (CFS), where its search method is optimized using particle swarm optimization (PSO). The procedure for feature selection is further discussed in Section 3.3.

In the second phase, a two-tier ensemble is formed. This phase is in charge of constructing a classification model via the mixture of three homogeneous ensembles, i.e., random forest (RF), gradient boosting machine (GBM), and extreme gradient boosting machine (XGBoost). These ensembles are stacked to produce a final prediction. According to this structure, other individual classifiers can also be considered, such as decision tree/J48 (DT) [37], random tree (RT) [38], and classification and regression tree (CART) [39]. Our objective is to benchmark our proposed classifier and the base classifiers that build the model. Besides, other individual classifiers, i.e., DT, RT, and CART, are included since RF is an improved version of RT, while GBM is built by an ensemble of CART. In addition, DT is a well-known algorithm that can be considered a representation of tree-based classifiers. The classification analysis and performance comparisons presented in Section 4 are based on the classification algorithms as mentioned above.

Finally, in the third phase, the proposed two-tier ensemble is assessed. The evaluation procedure is built upon *k*-fold cross validation, where *k* is set to 10. This procedure is also known as 10-fold cross validation (10CV).

Furthermore, three performance measures are typically employed in the imbalanced data problem. These are accuracy, *F*_1_, and area under ROC (AUC). Section 4 presents the experimental results of the paper.

### 3.3. Feature Selection

As we have mentioned above, some irrelevant input features might lower the classifier's performance. Hence, choosing an accurate and rigorous subset of features from a particular set of features for the prediction task is very challenging. In this paper, we exploit a correlation-based feature selection (CFS) as it is a widely known attribute evaluator for machine learning. Besides, in many cases, CFS gave comparable performance to the wrapper method and, in general, outperformed the wrapper method on small datasets [32]. It evaluates the relevance of a feature subset using information gain and entropy [32]. More specifically, insignificant and unnecessary features are omitted in this phase. Furthermore, we leverage an optimization technique, namely, particle swarm optimization (PSO), as a search technique. A number of experiments are carried out by varying the number of particles. The best feature set is then chosen by the maximum accuracy of the credal decision tree (CDT) classifier [40]. CDT uses imprecise probabilities and uncertainty measures for the splitting criterion. The performance of CDT is evaluated using *subsampling*, where the training set *D*_train_ is simply derived from a dataset *D*. The remaining part (*D*_test_) is used for testing. The procedure is then repeated *k* 260 times. In this work, we consider a sampling ratio of 80*/*20 and *k* = 50.

### 3.4. Classification Techniques

The proposed two-tier ensemble is built upon three different classifier ensembles, i.e., RF, GBM, and XGBoost in a stacked (parallel) manner.

Compared to conventional classifier ensembles that always exploit weak individual learners, in this work, we take into account strong ensemble learners as the base classifiers. The best learning *hyperparameters* of each base classifier are obtained using *grid* search by trying out all possible values. The area under ROC (AUC) [41] is employed as a stopping metric of the search. We briefly explain the three base classifiers used in this study as follows.

#### 3.4.1. Random Forest (RF)

This classifier takes bagging of decision tree procedure to evoke a large collection of trees to improve performance. Compared to other similar ensembles, RF requires less *hyperparameter* tuning. Original bagging decision tree yields tree-mutuality, which suffers from the effect of high variance. Hence, RF offers a variance reduction by introducing more randomness into the tree-generation procedure [29]. After performing *grid* search, optimized learning parameters of RF are *n*trees = 10,000, max depth = 9, min rows = 8, *n*bins = 256, *n*bins cats = 4096, sample rate = 0.56, histogram type = ^“^QuantilesGlobal^”^, and distribution = ^“^multinomial^”^.

#### 3.4.2. Gradient Boosting Machine (GBM)

This algorithm builds an ensemble of trees in a serial approach, where a weak model, e.g., a tree with only a few splits, is trained first and consecutively improves its performance by maintaining to generate new trees. Each new tree in the sequence is responsible for repairing the previous prediction error [30]. Based on the *grid* search, we set the learning parameters as follows: *n*trees = 10,000, max depth = 14, min rows = 1, *n*bins = 1024, *n*bins cats = 64, learn rate = 0.05, learn rate annealing = 0.99, distribution = ^“^bernoulli^”^, sample rate = 0.32, col sample rate = 0.97, and histogram type = ^“^QuantilesGlobal^”^.

#### 3.4.3. Extreme Gradient Boosting Machine (XGBoost)

Besides GBM, XGBoost [31] is another implementation of a gradient boosting algorithm. A wide variety of problems can be solved using gradient boosting. The rationale of the algorithm is to seek the fine-tuned learning parameters iteratively in order to reduce a cost function. Concerning computational efficiency (e.g., memory utilization and processor cache), XGBoost is better than GBM. Furthermore, it harnesses a more regularized model, thus minimizing the model complexity and improving predictive accuracy. We set the same *hyperparameter* settings as GBM. This work takes into account a two-tier ensemble, in which the abovementioned homogeneous ensembles are blended in a stacked approach. In practice, there are several combinations of base classifiers can be made. However, since we aim to prove the effectiveness of such architecture for CHD prediction, we consider those three ensembles as base classifiers. First, base classifiers are trained using the specified training set; then, a meta classifier, e.g., generalized linear model (GLM), is trained to predict the outcome. A two-tier ensemble consists of the following procedures:

- Train each of the C tier-1 ensembles (with the best *hyperparameter* settings) on the training set
- Perform 10CV on each ensemble and gather the prediction outcomes, *O*_1_, *O*_2_, *…*,*O*_*C*_
- The *M* prediction result values from each of the *C* ensembles are fused in such a way that a matrix *M* × *C* is formed. Together with original response vector *y*, train the meta classifier on the level-one data, *y* = *f* (*M* × *C*).
- Generate all label predictions from each tier-1 ensemble, feed into the meta classifier, and acquire the final tier-2 ensemble label prediction

## 4. Results

In this section, the results of all experiments are discussed. We firstly present the results of feature selection, followed by a classification analysis of CHD detection. In the end, this section benchmarks the proposed approach with existing ones. All experiments were performed on a Linux machine, 32 GB memory, and Intel Xeon processor. We used an open-source data mining tool, Weka [42], for feature selection, while the classification process for the CHD detection model was implemented in *R* with *H*_2_*O* package [43].

### 4.1. Results of Feature Selection

**Figure 2:**
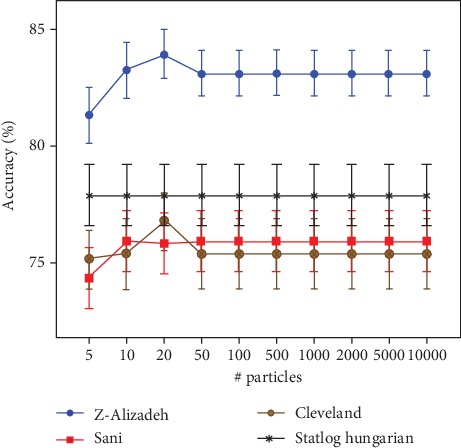
Classification accuracy of CDT for each CHD dataset w.r.t various number of particles.

First of all, we discuss the experiment of choosing the best feature set by running different numbers of particles in PSO. Parameter settings for PSO are depicted in Table 3. The results for the predictive accuracy of the CDT classifier are presented in Figure 2. It is clear that PSO with 20 particles is the best prediction performance on the Z-Alizadeh Sani dataset with an accuracy of 83.905 ± 1.036%. This trial produces a set of 27 features. The same number of particles has brought the best classification accuracy on the Statlog dataset. A set of 8 features are generated in this case with a predictive accuracy of 76.822 ± 1.241%. Furthermore, the best classification result of Cleveland dataset can be achieved with several numbers of particles, e.g., ten particles or 50-10,000 particles. In this case, seven significant features have been obtained. Surprisingly, in the Hungarian dataset, the different number of particles has not affected the classification accuracy as well as the selected features. Overall, the implementation of PSO, in which the number of particles is more than 50, does not bring a substantial change in the performance of CDT as well as the number of selected features (see Table 4).  Table 5 summarizes a set of input features obtained from the proposed approach for each dataset.

### 4.2. Coronary Heart Disease Classification Analysis

This section benchmarks the performance of a two-tier ensemble towards other classifiers, i.e., RF, GBM, XGBoost, DT, RT, and CART. We employ the mean AUC metric from 10CV. We also consider the results of the significance comparison among classifiers using a two-step statistical test. Following the recommendation of [28], an omnibus test using Friedman rank [44] and Iman-Davenport [45] are implemented. If the performance differences of classifiers can be detected, a Friedman post hoc test is undertaken. The Friedman test evaluates the hierarchy of the benchmarked classifiers, while Iman-Davenport figures out whether at least one classifier possesses a significant difference against others. Once such a difference is discovered; a pair-wise test using the Friedman post hoc with the associated *p* value is performed for multiple comparisons. Concerning the Friedman post hoc test, a comparison with a *control* is considered. To do so, the proposed algorithm is picked as a control classifier 360 for being benchmarked against other classifiers, e.g., DT, RT, CART, RF, GBM, and XGBoost. The indication of a significant difference is appraised by a *p* value that must be lower than the threshold (0.05 in our case).  Table 6 presents the mean of the AUC value and Friedman average rank, as well as the *p* result of the Iman-Davenport test. It should be pointed out that the lower the rank of the classifier, the better the classifier.

## 5. Discussion

Table 6 provides us evidence that the proposed algorithm arises as the best method, resulting from the fact that it is involved with the lowest rank. The *p* value = 3.69*E*-10 which denotes a significant difference (*p* < 0.05) in at least two benchmarked classifiers is found. This means the null hypothesis that implies a commensurate performance among all classifiers can be rejected. Furthermore, as the null hypothesis is rejected, we estimate the performance differences of the pairs using the Friedman post hoc test. In this case, the best performing classifier (proposed algorithm) is chosen as a control classifier since it possesses the lowest mean rank.  Table 7 depicts the results of the statistical comparison among the pairs. It demonstrates how the proposed algorithm surpasses other individual classifier's families, i.e., DT, RT, and CART, and other classifier ensemble's family, i.e., RF, GBM, and XGBoost. It worth mentioning that the performance differences between the proposed algorithm and all other individual classifiers are highly significant (*p* < 0.05), yet the performance difference between the proposed algorithm and all other classifier ensembles is not too significant (*p* > 0.05).

For the sake of universality and comprehensiveness, we contrast the proposed algorithm with current existing studies that have utilized the four datasets, i.e., Z-Alizadeh Sani, Statlog, Cleveland, and Hungarian, in their experiment. Tables 8–11 summarize the results for each dataset in terms of three performance measures, i.e., accuracy, *F*_1_, and AUC. Generally speaking, the proposed algorithm has outperformed the most recent approaches applied on Z-Alizadeh Sani, Statlog, and Hungarian datasets such as support vector machine (SVM) [25, 46], logistic regression [23], voting-based ensemble [26], and neural network [21]. Our proposed approach is still comparable against some approaches applied to the Cleveland dataset. It performs better than SMO-based expert system [15] in terms of *F*_1_ metric. The best classification accuracy on the Cleveland dataset still goes for [14]; however, it should be noted that [14] used one round of hold-out without replication which is less reliable than 10CV.

## 6. Conclusion

In this study, we proposed an improved detection model of coronary heart disease (CHD) based on a two-tier ensemble. The proposed method was built by the stacking of three different ensemble learners, such as the random forest, gradient boosting machine, and extreme gradient boosting machine. The proposed detection model was tested on four different publicly available datasets, i.e., Z-Alizadeh Sani, Statlog, Cleveland, and Hungarian, in order to provide a fair benchmark against existing studies. We also conducted a two-step statistical significance test to evaluate the performance significance among benchmarked classifiers, where it currently lacks in the current literature. Based on the experimental results, our proposed model was able to outperform state-of-the-art CHD detection methods with respect to accuracy, *F*_1_, and AUC value. The results reflected the highest result obtained so far applied to those aforementioned datasets.

## Figures and Tables

**Table 1:** Summarization of existing methods for CHD prediction in chronological order.

Study	Technique	Feature selection	Validation method	Dataset
Ozcift and Gulten [13]	Rotation forest	No	10CV	Cleveland
Muthukaruppan and Er [14]	Fuzzy expert system	No	Hold-out	Cleveland
Nahar et al. [15]	SMO	Yes	10CV	Cleveland
Alizadehsani et al. [16]	Bagging-C4.5	Yes	10CV	Z-Alizadeh Sani
Alizadehsani et al. [17]	SMO	Yes	10CV	Z-Alizadeh Sani
Alizadehsani et al. [18]	SVM	Yes	10CV	Z-Alizadeh Sani
Verma et al. [19]	MLP	Yes	10CV	Cleveland, IGMC
Qin et al. [20]	EA-MFS	Yes	10CV	Z-Alizadeh Sani
Arabasadi et al. [21]	Hybrid NN-GA	Yes	10CV	Z-Alizadeh Sani, Cleveland, Hungarian, Long-beach-va, and Switzerland
Haq et al. [22]	SVM	Yes	10CV	Cleveland
Dwivedi [23]	Logistic regression	No	10CV	Statlog
Ahmadi et al. [24]	NN	Yes	Hold-out	Cleveland
Abdar et al. [25]	SVM	Yes	10CV	Z-Alizadeh Sani
Raza [26]	Voting ensemble	No	10CV	Statlog
Amin et al. [9]	Voting ensemble	Yes	10CV	Cleveland, Statlog
Mohan et al. [27]	HRFLM	No	Notmentioned	Cleveland, Hungarian, Long-beach-va, and Switzerland

**Table 2:** Summarization of each dataset's characteristics and properties.

Dataset	# features	# instances	Ratio between normal and CHD
Z-Alizadeh Sani	54	303	1 : 2.5
Statlog	13	261	1 : 0.78
Cleveland	13	303	1 : 0.85
Hungarian	13	294	1 : 0.56

**Table 3:** Parameter settings used in particle swarm optimization-based feature selection.

Parameter	Value
*c*_1_	1.0
*c*_2_	2.0
Maximum generations	30
Number of particles	5, 10, 20, 50, 100, 500, 1000, 2000, 5000, and 10000
Mutation type	Bit-flip
Mutation probability	0.01
Prune	False

**Table 4:** Number of selected features for each CHD dataset w.r.t different number of particles.

# particles	Z-Alizadeh	# selected features		Hungarian
		Cleveland	Statlog	
5	15	10	10	6
10	26	7	7	6
20	27	9	8	6
50	13	7	7	6
100	13	7	7	6
500	13	7	7	6
1000	13	7	7	6
2000	13	7	7	6
5000	13	7	7	6
10000	13	7	7	6

**Table 5:** The selected features obtained from PSO based feature selection for each dataset.

Dataset	# selected features	Feature name
Z-Alizadeh Sani	27	Age, hypertension, airway disease, thyroid disease, congestive heart failure, dyslipidemia, blood pressure, systolic murmur, diastolic murmur, typical chest pain, dyspnea, atypical, nonanginal, low threshold angina, ST elevation, T inversion, poor R progression, fasting blood sugar, LDL, HDL, blood urea nitrogen, erythrocyte sedimentation rate, white blood cell, neutrophil, ejection fraction, region with regional wall motion abnormality, and valvular heart disease.
Statlog	8	Gender/sex, chest pain type, resting electrocardiographic results, maximum heart rate achieved, exercise induced angina, ST depression, number of major vessels, and thallium stress test result.
Cleveland	7	Chest pain type, resting electrocardiographic results, maximum heart rate achieved, exercise induced angina, oldpeak, number of major vessels, and thallium stress test result.
Hungarian	6	Gender/sex, chest pain type, heart rate, old peak, slope, and number of major vessels.

**Table 6:** Results of mean value of AUC (%) and the Friedman rank and Iman-Davenport tests (the best value is indicated in bold).

Algorithm	Z-Alizadeh Sani	Statlog	Cleveland	Hungarian	Friedman rank	Iman-Davenport *p* value
DT	76.30	80.30	79.80	77.10	5.50	3.69E-10
RT	69.90	78.90	75.20	73.60	7.00	
CART	78.20	79.80	78.60	80.30	5.50	
RF	92.47	89.49	**90.94**	91.54	1.75	
GBM	88.99	88.13	89.24	91.13	2.75	
XGBoost	87.65	80.73	85.30	86.98	4.00	
**Proposed**	**99.70**	**93.42**	85.86	**92.98**	**1.50**	

**Table 7:** Comparative results of all classifiers of the w.r.t Friedman post hoc test.

Comparison	Friedman post hoc *p* value
Proposed vs. DT	0.0088
Proposed vs. RT	0.00031
Proposed vs. CART	0.0088
Proposed vs. RF	0.86
Proposed vs. GBM	0.41
Proposed vs. XGBoost	0.10

**Table 8:** Comparison of the proposed method with some prior studies using the Z-Alizadeh Sani dataset (the best value is indicated in bold).

Study	Technique	# of features	Validation method	Accuracy	(%)	*F*_1_ (%)	AUC (%)	Statistical test
[16]	Bagging-DT	20	10CV	79.54 61.46 and (RCA)	(Lad), (LCX), 68.96	Not reported	Not reported	No
[17]	Information gain-SMO	34	10CV	94.08		Not reported	Not reported	No
[18]	Information gain-SVM	24	10CV	86.14 83.17 and 83.5	(Lad), (LCX), (RCA)	Not reported	Not reported	No
[21]	Neural network genetic algorithm	22	10CV	93.85		Not reported	Not reported	No
[20]	Ensemble algorithm multiple feature selection	34	10CV	93.70		95.53	Not reported	No
[46]	Support vector machine feature engineering	32	10CV	96.40		Not reported	Not reported	No
[25]	*ν*-support vector machine	29	10CV	93.08		91.51	Not reported	No
This paper	Two-tier ensemble PSO-based feature selection	27	10CV	**98.13**		**96.60**	**98.70**	Two-step statistical test

**Table 9:** Comparison of the proposed method with some prior studies using the StatLog dataset (the best value is indicated in bold).

Study	Technique	# of features	Validation method	Accuracy (%)	*F*_1_ (%)	AUC (%)	Statistical test
[23]	Logistic regression	13	10CV	85	87	Not reported	No
[26]	Ensemble voting logistic regression multilayer perceptron naive Bayes	13	10CV	89	87	88	No
This paper	Two-tier ensemble PSO-based feature selection	8	10CV	**93.55**	**91.67**	**93.42**	Two-step Statistical test

**Table 10:** Comparison of the proposed method with some prior studies using the Cleveland dataset (the best value is indicated in bold).

Study	Technique	# of features	Validation method	Accuracy (%)	*F*_1_ (%)	AUC (%)	Statistical test
[13]	Rotation forest-J48-CFS	7	10CV	84.48	Not reported	**89.5**	No
[14]	PSO fuzzy expert systems	76	Hold-out	**93.27**	Not reported	Not reported	No
[15]	SMO-expert-based feature selection	8	10CV	84.49	86.2	Not reported	No
[19]	CFS-PSO-clustering-MLP	5	10CV	90.28	Not reported	Not reported	No
[22]	Logistic regression-LASSO	6	10CV	89	Not reported	Not reported	No
[24]	Boosted-C5.0 and neural network	12	10CV	77.8 & 81.9	Not reported	Not reported	Paired *t* test
[9]	Voting-naive Bayes-logistic regression	9	10CV	87.41	Not reported	Not reported	No
This paper	Two-tier ensemble PSO-based feature selection	7	10CV	85.71	**86.49**	85.86	Two-step statistical test

**Table 11:** Comparison of the proposed method with some prior studies using the Hungarian dataset (the best value is indicated in bold).

Study	Technique	# of features	Validation method	Accuracy (%)	*F*_1_ (%)	AUC (%)	Statistical test
[21]	Neural network genetic algorithm	14	10CV	87.1	Not reported	Not reported	No
This paper	Two-tier ensemble PSO-based feature selection	6	10CV	**91.18**	**90.91**	**92.98**	Two-step statistical test

## Data Availability

The link of datasets used to support the findings of this study are included within the article.
